# Supplementary material for: Selection signatures and population dynamics of transposable elements in lima bean
Source: Commun Biol. 2023 Aug 2;6:803. doi: 10.1038/s42003-023-05144-y (PMC10397206; doi:10.1038/s42003-023-05144-y)
Supplement: Supplementary file 2 — Supplementary Materials [file 42003_2023_5144_MOESM2_ESM.pdf]

Supplementary materials for:

Selection signatures and population dynamics of transposable elements in lima bean

Daniela Lozano-Arce[1], Tatiana García[2], Laura Natalia Gonzalez-Garcia[1,3], Romain Guyot[3], Maria Isabel Chacón-Sánchez[2,+], Jorge Duitama[1,*,+]

[1]Systems and Computing Engineering Department. Universidad de los Andes, Bogotá, Colombia
[2]Departamento de Agronomía, Facultad de Ciencias Agrarias, Universidad Nacional de Colombia, Bogotá, Colombia.
[3]Institut de Recherche pour le Développement (IRD), UMR DIADE, Université de Montpellier, CIRAD, 34394 Montpellier, France

* Corresponding author: ja.duitama@uniandes.edu.co
+ These authors jointly supervised this work

Supplementary data (Separate files)

Supplementary Data 1. Database of transposable elements annotated in the lima bean V1 reference genome.
Supplementary Data 2. Database of transposable elements annotated in the common bean reference genome V1.
Supplementary Data 3. CIAT seed passport information for the 60 accessions sequenced in this study.
Supplementary Data 4. Genes observed in the Andean and Mesoamerican gene pools within selective sweeps identified by at least one of three approaches.
Supplementary Data 5. Matrix of TEs with PAV in lima bean.
Supplementary Data 6. TEs with PAV that differentiate Andean and Mesoamerican gene pools.
Supplementary Data 7. TEs with PAV close to genes reported to be important in lima bean domestication.
Supplementary Data 8. Numeric supporting data for figures

Supplementary Tables

Supplementary Table 1. Summary of the lima bean and common bean transposon database. Span values are shown in Mega base pairs (Mbp)

Class	Family	Garcia et al., 2021		This work			
		P. lunatus		*P. lunatus*		*P. vulgaris*	
		Number	***Span***	***Number***	***Span***	***Number***	***Span***
**Class 1**							
**LTR retrotransposons**							
	LTR (Unclassified)	47143	15.32	2462	1.99	4225	8
	Copia (Unclassified)	47606	34.4	4271	4.93	4835	6.57
	Copia/ALE-RETROFIT	0	0	2866	4.68	4711	6.25
	Copia/IVANA-ORYCO	0	0	1966	2.78	1980	2.63
	Copia/SIRE	0	0	11402	22.7	14291	25.03
	Copia/TAR-TORK	0	0	3126	5.56	3903	5.21
	Gypsy (Unclassified)	293946	148.72	9448	10.27	9175	11.32
	Gypsy/ATHILA	0	0	3575	5.01	3072	4.36
	Gypsy/CRM	0	0	12529	23.58	12542	18.62
	Gypsy/REINA	0	0	687	1.09	858	1.24
	Gypsy/TAT	0	0	26116	53.07	38655	78.01
	Gypsy/TEKAY-DEL	0	0	25544	75.11	3518	5.08
	TRIM	7726	1.5	5727	1.37	4216	0.99
**Non-LTR retrotransposons**							
	LINE	27191	8.93	15034	6.86	19221	10.21
	SINE	2591	0.3	614	0.11	628	0.11
**Class 2**							
**DNA transposons**							
	Helitron	15229	5.55	21589	7.36	17986	6.25
	CACTA	41461	15.15	25678	13.2	27355	11.43
	Harbinger	550	0.38	429	0.35	344	0.23
	hAT	3013	0.88	964	0.41	2059	0.9
	MULE	17825	5.54	10299	4.19	12083	5.09
	DTA	0	0	2590	0.66	4710	1.16
	DTC	0	0	13926	3.95	16761	4.75
	DTH	0	0	1298	0.29	465	0.11
	DTM	0	0	11555	3.18	11421	2.88
	DTT	0	0	767	0.19	1240	0.34
**MITE transposons**							
	DTA	0	0	1043	0.19	2299	0.46
	DTC	0	0	446	0.11	1002	0.17
	DTH	0	0	235	0.04	221	0.03
	DTM	0	0	7332	1.36	6387	1.29

	DTT	0	0	171	0.03	137	0.01
**Pararetrovirus**							
				91	0.11	-	0
**Total**		**504281**	**236.67**	**223780**	**254.73**	**230300**	**218.73**

Supplementary Table 2. Counts per chromosome for genes observed within selected windows detected in the Andean and Mesoamerican gene pools identified by three different approaches.

	**XP-CLR**		**50 Kbp window**		**Gene diversity**	
**Chromosome**	**Andean**	**Mesoamerican**	**Andean**	**Mesoamerican**	**Andean**	**Mesoamerican**
Pl01	142	168	84	255	21	110
Pl02	76	130	339	498	102	206
Pl03	152	164	309	217	82	111
Pl04	95	72	121	400	59	191
Pl05	90	82	183	16	47	11
Pl06	61	70	217	137	64	88
Pl07	133	162	254	237	71	123
Pl08	97	117	151	26	50	30
Pl09	155	141	222	20	95	11
Pl10	56	59	152	1	51	15
Pl11	125	113	231	200	52	85
**Total**	**1182**	**1278**	**2263**	**2007**	**694**	**981**

Supplementary Table 3. Counts of TE deletion events for each sample.

Accession ID	TE Deletions	Population
G25955	9595	DOM_AI
G25898	8891	DOM_AI
G25900	9564	DOM_AI
G26709	9020	DOM_AI
G25897	8580	DOM_AI
G25540	9323	DOM_AI
G25981	9185	DOM_AI
G26661	9128	DOM_AI
G25987	9148	DOM_AI
G26659	9013	DOM_AI
G25172	8658	DOM_AI
G25910	9168	DOM_AI
G26184	7577	DOM_AI
G26703	9690	DOM_AI
G26464	9397	WILD_AI
G25915	8863	WILD_AI
G26463	8918	WILD_AI
G26609	8992	WILD_AI
G26607	9307	WILD_AI
G26348	9674	WILD_AI
G26465	9305	WILD_AI
G26467	8450	WILD_AI
G26468	10022	WILD_AI
G26751	9323	WILD_AI
G25914	9047	WILD_AI
G25916	9875	WILD_AI
G26469	8312	WILD_AI
G26460	9321	WILD_AI
G26608	1678	WILD_AI
G27429	328	DOM_MI
G26529	1396	DOM_MI
G25815D	835	DOM_MI
G26315	1787	DOM_MI
G26323	2120	DOM_MI
G26444	1686	DOM_MI
G27424	535	DOM_MI
G25293	2749	DOM_MI
G26717	1125	DOM_MI
G27445	1527	DOM_MI

G27422	2196	DOM_MI
G25811	1868	DOM_MI
G25254	1515	DOM_MI
G27423	714	DOM_MI
G27419	890	DOM_MI
G25400	1925	DOM_MI
G25514	1651	DOM_MI
G27435	3526	DOM_MI
G27455	431	DOM_MI
G26518	3871	WILD_MI
G26358	2894	WILD_MI
G25232	3409	WILD_MI
G25231	4446	WILD_MI
G26742	2500	WILD_MI
G26517	2758	WILD_MI
G26741	3806	WILD_MI
G25970	1954	WILD_MI
G26360	5321	WILD_MI
G26256	2434	WILD_MI
G25230	3291	WILD_MI
G26680	5664	WILD_MI
G25229	4582	WILD_MI

Supplementary Table 4. Wilcoxon test of significance between pairs of populations.

**Pairwise comparison**	**P-value**
WILD_AI / DOM_AI	0.6487
WILD_AI / WILD_MI	0.002409
WILD_MI / DOM_MI	0.1142
DOM_AI / DOM_MI	0.01298
WILD_AI / DOM_MI	0.005044
WILD_MI / DOM_AI	0.003124

Supplementary Table 5. TEs with PAV that differentiate Wild and Domesticated Mesoamerican gene pools.

Chr	First	Last	Length	Taxonomy
Pl01	30120973	30130866	9893	LTR/Gypsy/TEKAY-DEL
Pl01	32533226	32536111	2885	LTR/Gypsy/TEKAY-DEL
Pl02	4167756	4173830	6074	LTR/Gypsy/TAT
Pl02	4183603	4184106	503	LTR/Copia
Pl02	4191229	4192516	1287	LTR/Gypsy/ATHILA
Pl02	23625003	23634624	9621	LTR/Gypsy/TEKAY-DEL
Pl02	32495121	32499189	4068	LTR/Copia/ALE-RETROFIT
Pl03	4474021	4475488	1467	LTR/Copia/SIRE
Pl03	30500889	30501926	1037	LTR/Copia/SIRE
Pl03	30501927	30508858	6931	LTR/Gypsy/TEKAY-DEL
Pl04	41280688	41284907	4219	LTR/Gypsy/TEKAY-DEL
Pl04	42268085	42269233	1148	LTR/Copia/SIRE
Pl05	15412917	15422818	9901	LTR/Gypsy/TEKAY-DEL
Pl05	32262582	32264953	2371	LTR/Gypsy/TEKAY-DEL
Pl06	21111098	21117971	6873	LTR/Copia/IVANA-ORYCO
Pl07	24278407	24279558	1151	LTR/Gypsy/CRM
Pl07	24553514	24557171	3657	LTR/Gypsy/TEKAY-DEL
Pl07	24557173	24558876	1703	LTR/Gypsy/TEKAY-DEL
Pl07	24557385	24561397	4012	LTR/Gypsy/TAT
Pl07	24559890	24561582	1692	LTR/Gypsy/TEKAY-DEL
Pl08	3310978	3317425	6447	DNA/CACTA
Pl08	14913826	14914825	999	LTR/Copia/SIRE
Pl08	14914888	14915555	667	LINE
Pl08	14915265	14915876	611	LINE
Pl08	14917442	14918102	660	LTR/Gypsy/CRM
Pl08	14923682	14924182	500	DNA/Helitron
Pl09	6883178	6886724	3546	LTR/Gypsy/TEKAY-DEL
Pl09	6886949	6892493	5544	LTR/Gypsy/TEKAY-DEL
Pl09	6890105	6892526	2421	LTR/Gypsy/TEKAY-DEL
Pl09	8020797	8026512	5715	LTR/Copia/TAR-TORK
Pl09	9636070	9637391	1321	LTR/Gypsy/TEKAY-DEL
Pl09	9636282	9638577	2295	LTR/Gypsy/TAT
Pl09	17614351	17618674	4323	LTR/Copia/SIRE
Pl10	31323376	31326096	2720	LTR/Gypsy/TEKAY-DEL
Pl10	31327859	31337864	10005	LTR/Gypsy/TEKAY-DEL
Pl10	44249663	44250940	1277	LTR/Gypsy/TEKAY-DEL
Pl10	44255057	44255679	622	LTR/Copia/SIRE
Pl10	44255787	44256391	604	LTR/Gypsy/ATHILA

Pl10	44256414	44257026	612	LTR/Copia
Pl10	44260048	44260624	576	DNA/DTC
Pl10	44261043	44261574	531	LTR/Gypsy
Pl10	44269420	44270332	912	DNA/hAT
Pl10	44270676	44271266	590	DNA/CACTA
Pl10	44272177	44273098	921	LTR/Gypsy/TAT
Pl10	44277244	44278014	770	LTR
Pl10	44278791	44279298	507	LTR/Gypsy/TEKAY-DEL
Pl10	44279298	44279888	590	DNA/MULE
Pl10	44280048	44280790	742	LTR/Gypsy/TEKAY-DEL
Pl10	44280095	44281013	918	LTR/Gypsy
Pl10	44280930	44282077	1147	LTR
Pl10	44282188	44283487	1299	LTR
Pl10	44285200	44287595	2395	LTR/Gypsy/TAT
Pl10	44292498	44293067	569	LTR/Gypsy
Pl10	44293355	44294146	791	LTR/Gypsy
Pl10	44298259	44299257	998	LTR/Copia/IVANA-ORYCO
Pl10	44299502	44300305	803	LTR/Copia/IVANA-ORYCO
Pl10	44535121	44540331	5210	LTR/Copia/TAR-TORK
Pl11	10639146	10639974	828	DNA/DTC
Pl11	18551328	18561389	10061	LTR/Gypsy/TEKAY-DEL
Pl11	21812957	21815311	2354	LTR/Gypsy/ATHILA
Pl11	47143736	47144291	555	LTR

Supplementary Table 6. Filtering sizes for the transposon superfamilies annotated for lima bean and Common bean.

**Superfamily/family**	**Filter regions (bp)**
LINE	<100
LTR/Copia	<500
LTR/Gypsy	<500
LTR/TRIM	<100
SINE	<100
DNA	<100
MITE	<50

Supplementary Table 7. Validation and count of presence-absence variants (PAV) of TEs.

		Variable Tes				Percentage			
**Taxonomy**	**Count PAV**	**100**	**95**	**90**	**85**	**100**	**95**	**90**	**85**
LTR	2462	357	362	369	381	14.50	14.70	14.99	15.48
Copia	4271	676	686	694	707	15.83	16.06	16.25	16.55
Copia/ALE-RETROFIT	2866	514	527	532	537	17.93	18.39	18.56	18.74
Copia/IVANA-ORYCO	1966	504	517	525	530	25.64	26.30	26.70	26.96
Copia/SIRE	11402	3350	3461	3509	3567	29.38	30.35	30.78	31.28
Copia/TAR-TORK	3126	850	880	901	918	27.19	28.15	28.82	29.37
Gypsy	9448	1379	1405	1438	1467	14.60	14.87	15.22	15.53
Gypsy/ATHILA	3575	824	835	849	859	23.05	23.36	23.75	24.03
Gypsy/CRM	12529	3727	3845	3917	4004	29.75	30.69	31.26	31.96
Gypsy/REINA	687	194	201	210	215	28.24	29.26	30.57	31.30
Gypsy/TAT	26116	8961	9246	9521	9767	34.31	35.40	36.46	37.40
Gypsy/TEKAY-DEL	25544	13195	13515	13585	13680	51.66	52.91	53.18	53.55
TRIM	5727	336	339	341	341	5.87	5.92	5.95	5.95
LINE	15034	1924	1945	1956	1969	12.80	12.94	13.01	13.10
SINE	614	54	54	54	54	8.79	8.79	8.79	8.79
Helitron	21589	3166	3181	3194	3209	14.66	14.73	14.79	14.86
DNA/CACTA	25678	3327	3343	3351	3366	12.96	13.02	13.05	13.11
DNA/Harbinger	429	69	69	72	73	16.08	16.08	16.78	17.02
DNA/hAT	964	101	102	102	104	10.48	10.58	10.58	10.79
DNA/MULE	10299	1080	1090	1097	1105	10.49	10.58	10.65	10.73
DNA/DTA	2590	352	355	356	358	13.59	13.71	13.75	13.82
DNA/DTC	13926	2061	2065	2077	2084	14.80	14.83	14.91	14.96
DNA/DTH	1298	114	114	115	115	8.78	8.78	8.86	8.86
DNA/DTM	11555	1012	1020	1025	1035	8.76	8.83	8.87	8.96
DNA/DTT	767	42	42	44	45	5.48	5.48	5.74	5.87
MITE/DTA	1043	54	54	55	56	5.18	5.18	5.27	5.37
MITE/DTC	446	98	100	100	101	21.97	22.42	22.42	22.65
MITE/DTH	235	30	30	30	31	12.77	12.77	12.77	13.19
MITE/DTM	7332	1510	1525	1536	1551	20.59	20.80	20.95	21.15
MITE/DTT	171	17	17	17	17	9.94	9.94	9.94	9.94
Pararetrovirus	91	30	30	30	30	32.97	32.97	32.97	32.97
	223780	49908	50955	51602	52276	18.03	18.35	18.60	18.85
	**Total**					**Average**			

Supplementary figures

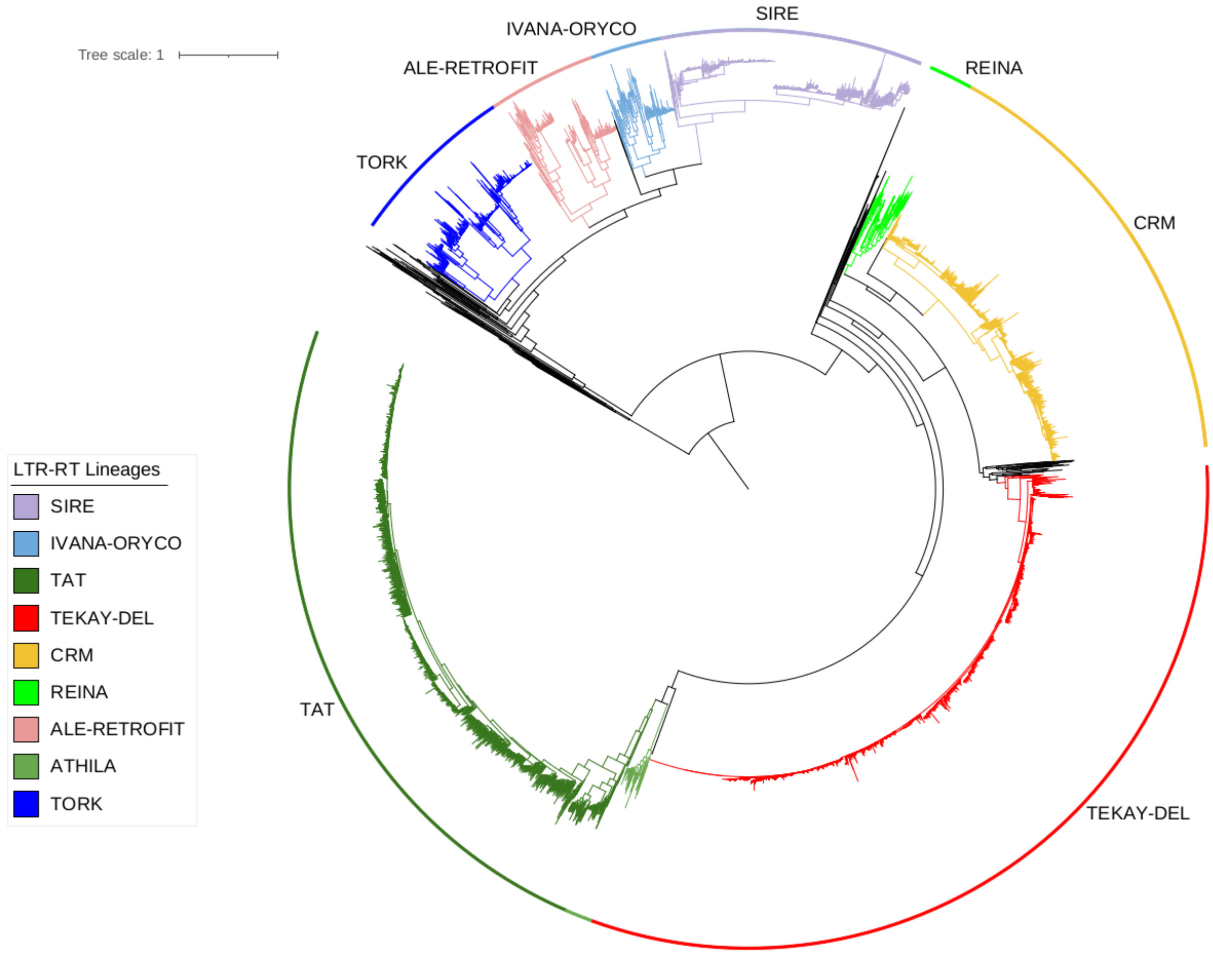

Supplementary Figure 1. Phylogenetic analysis of the *P. lunatus* LTR retrotransposon sequences. The unrooted phylogenetic tree of Gypsy (REINA, CRM, TAT, ATHILA and TEKAY-DEL) and Copia (TORK, ALE-RETROFIT, IVANA-ORYCO, SIRE) elements was constructed on the basis of the reverse-transcriptase domain of *P. lunatus.*

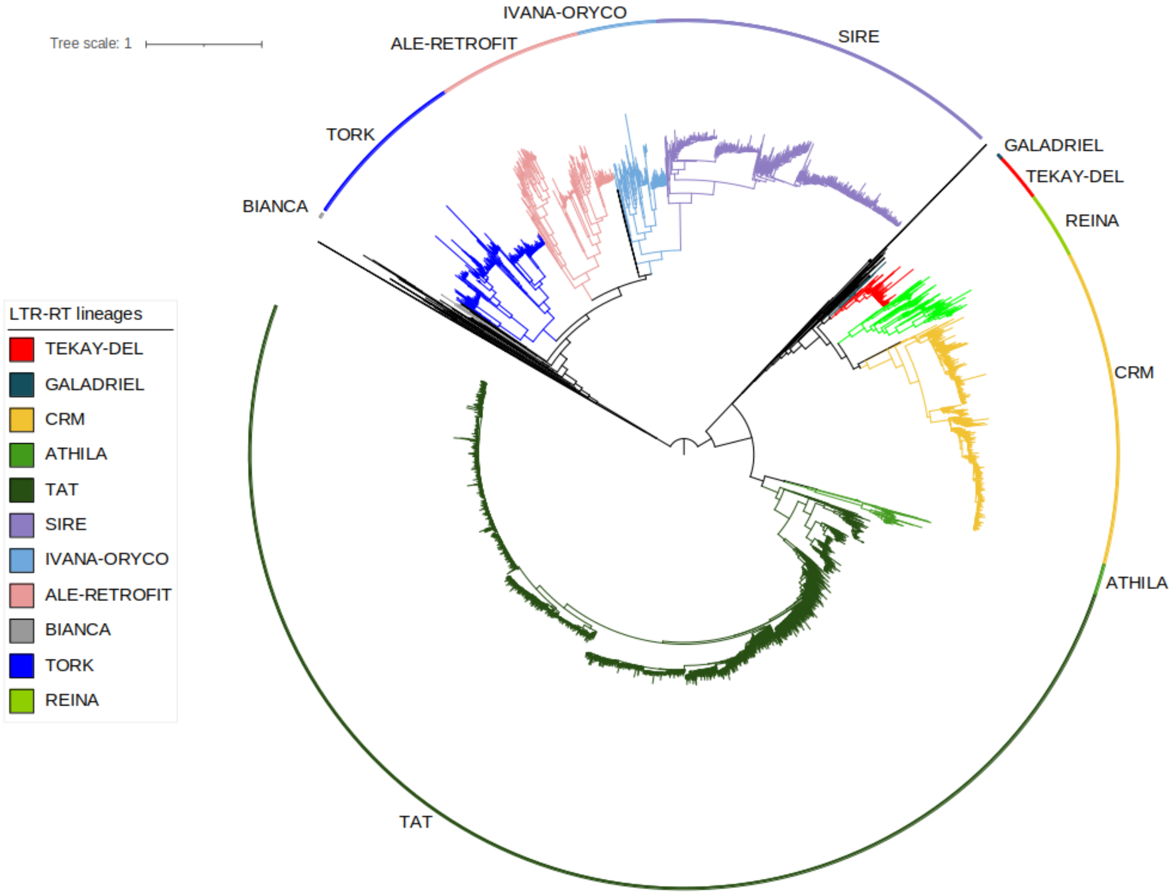

Supplementary Figure 2. Phylogenetic analysis of the *P. vulgaris* LTR retrotransposon sequences. The unrooted phylogenetic tree of Gypsy (REINA, CRM, TAT, ATHILA and TEKAY-DEL) and Copia (TORK, ALE-RETROFIT, IVANA-ORYCO, SIRE) elements was constructed on the basis of the reverse-transcriptase domain of *P. vulgaris.*

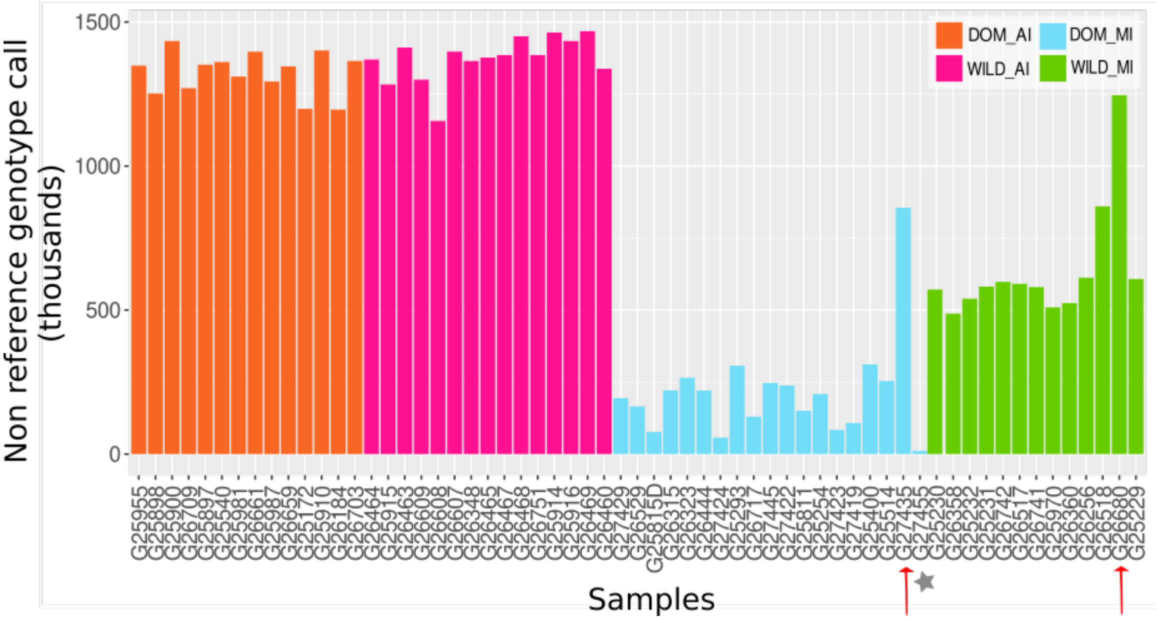

Supplementary Figure 3. Distribution of non homozygous-reference genotype calls per sequenced accession. Colors differentiate the population of origin for each accession (DOM_AI=domesticated Andean, WILD_AI= wild Andean, DOM_MI= domesticated Mesoamerican, WILD_MI= wild Mesoamerican). The accessions highlighted with a red arrow (G27435, G26680) are admixed between Mesoamerican gene pools (MI and MII) and the accession marked with a star corresponds to the reference genome.

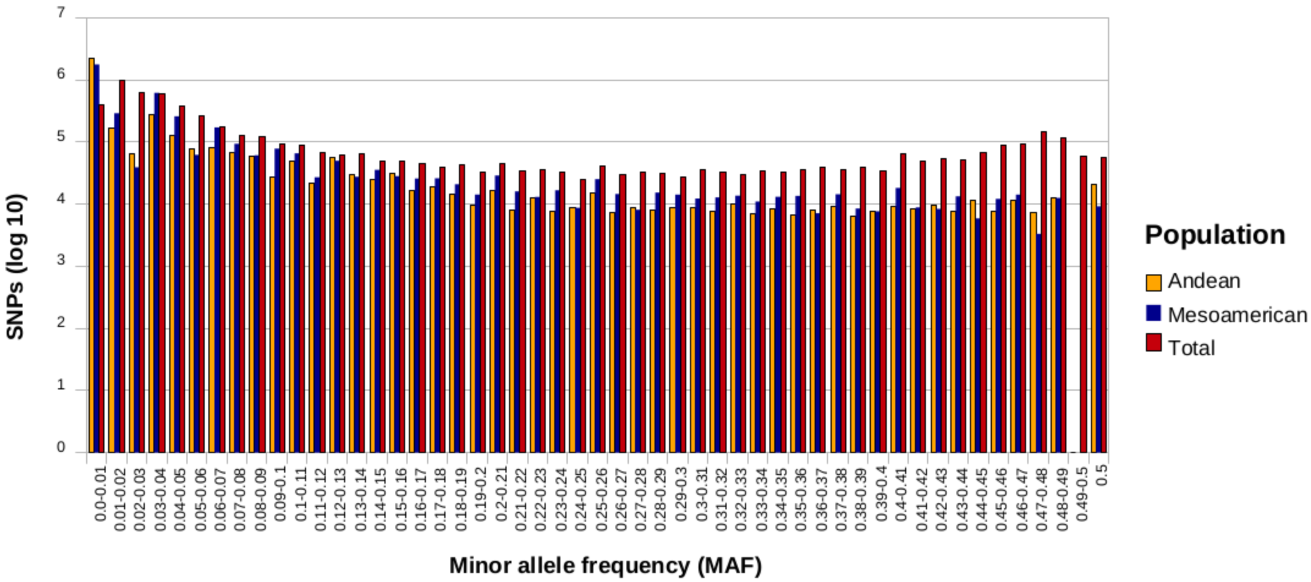

Supplementary Figure 4. Minor allele frequency of SNPs in the genomic variation database.

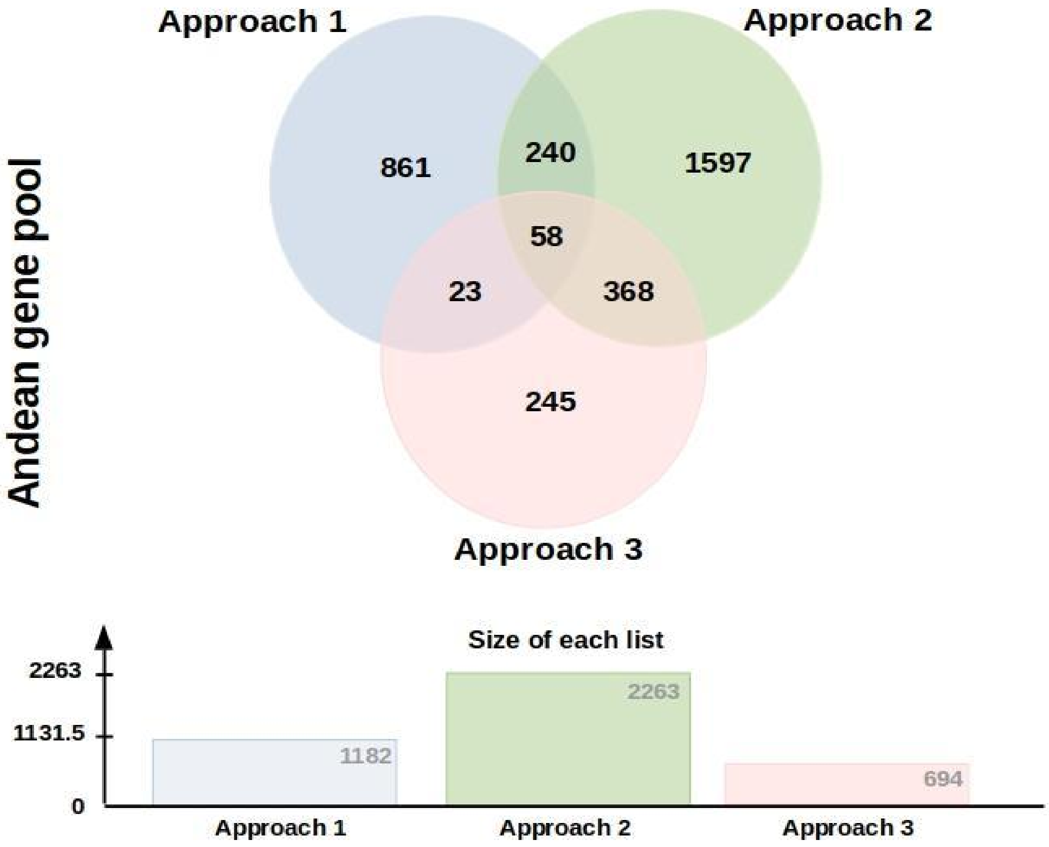

Supplementary Figure 5. Venn diagram of genes identified in selective sweeps in the Andean gene pool of lima bean. Approach 1: 50Kb/5Kb sliding-window approach using XP-CLR. Approach 2: 50Kb/5Kb sliding-window approach using popgenome. Approach 3: gene-by-gene analysis using NGSEP.

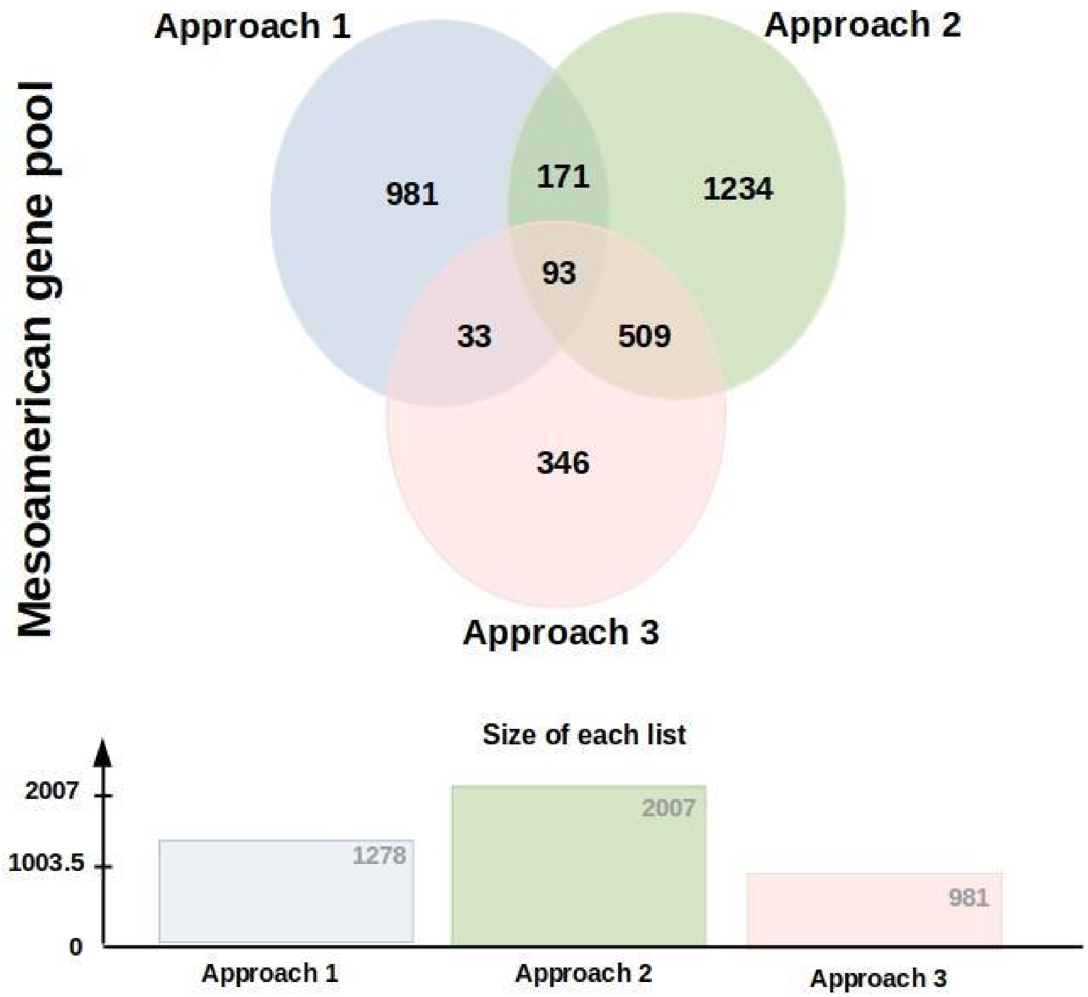

Supplementary Figure 6. Venn diagram of genes identified in selective sweeps in the Mesoamerican gene pool of lima bean. Approach 1: 50Kb/5Kb sliding-window approach using XP-CLR. Approach 2: 50Kb/5Kb sliding-window approach using popgenome. Approach 3: gene-by-gene analysis using NGSEP.

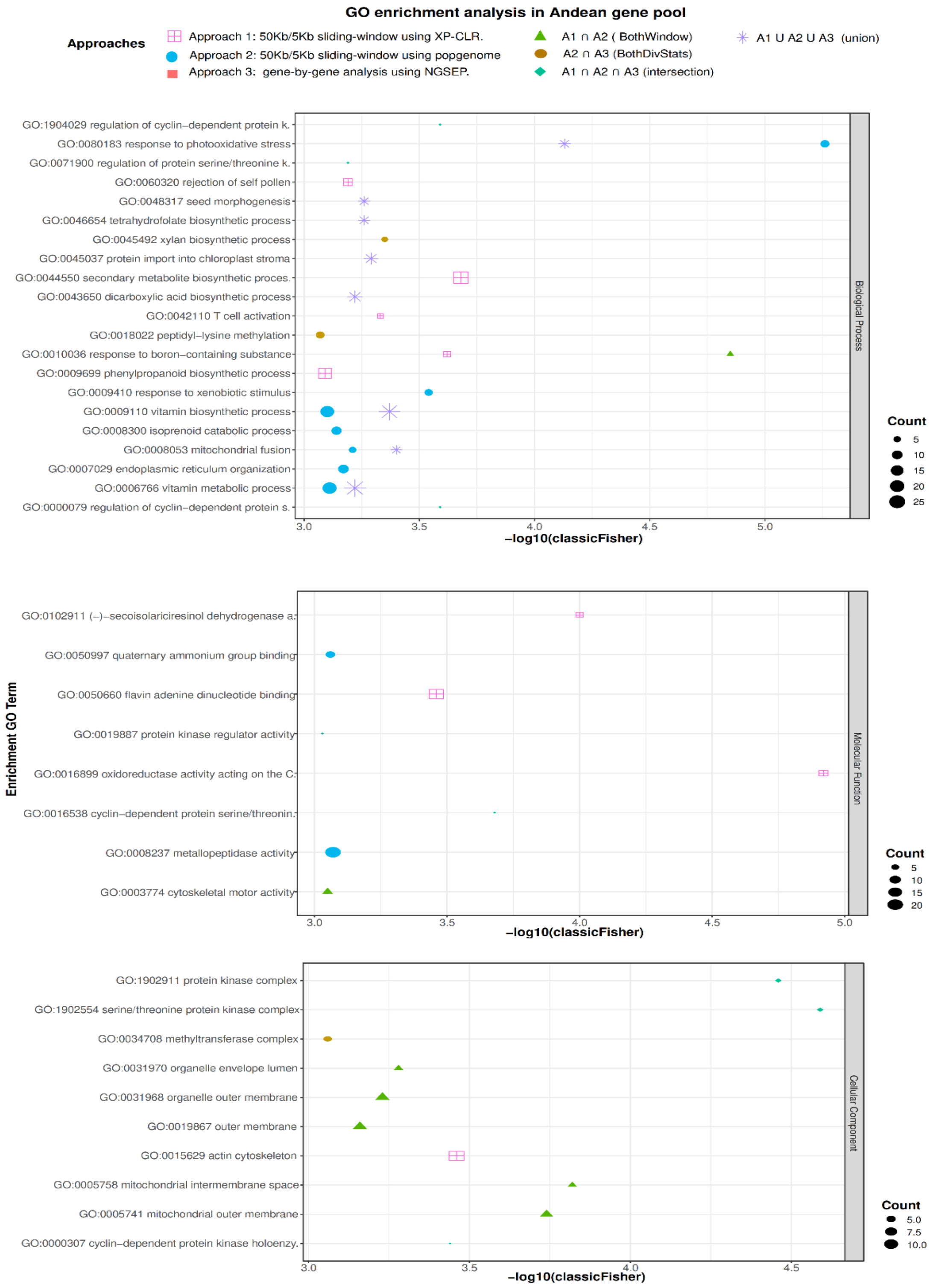
GO enrichment analysis in Andean gene pool
Approaches
Approach 1: 50Kb/5Kb sliding-window using XP-CLR.
Approach 2: 50Kb/5Kb sliding-window using popgenome
Approach 3: gene-by-gene analysis using NGSEP.
A1 ∩ A2 (BothWindow)
A2 ∩ A3 (BothDivStats)
A1 ∩ A2 ∩ A3 (intersection)
A1 U A2 U A3 (union)
GO:1904029 regulation of cyclin−dependent protein k.
GO:0080183 response to photooxidative stress
GO:0071900 regulation of protein serine/threonine k.
GO:0060320 rejection of self pollen
GO:0048317 seed morphogenesis
GO:0046654 tetrahydrofolate biosynthetic process
GO:0045492 xylan biosynthetic process
GO:0045037 protein import into chloroplast stroma
GO:0044550 secondary metabolite biosynthetic proces.
GO:0043650 dicarboxylic acid biosynthetic process
GO:0042110 T cell activation
GO:0018022 peptidyl−lysine methylation
GO:0010036 response to boron−containing substance
GO:0009699 phenylpropanoid biosynthetic process
GO:0009410 response to xenobiotic stimulus
GO:0009110 vitamin biosynthetic process
GO:0008300 isoprenoid catabolic process
GO:0008053 mitochondrial fusion
GO:0007029 endoplasmic reticulum organization
GO:0006766 vitamin metabolic process
GO:0000079 regulation of cyclin−dependent protein s.
Biological Process
Count
5
10
15
20
25
3.0
3.5
4.0
4.5
5.0
−log10(classicFisher)
Enrichment GO Term
GO:0102911 (−)−secoisolariciresinol dehydrogenase a.
GO:0050997 quaternary ammonium group binding
GO:0050660 flavin adenine dinucleotide binding
GO:0019887 protein kinase regulator activity
GO:0016899 oxidoreductase activity acting on the C.
GO:0016538 cyclin−dependent protein serine/threonin.
GO:0008237 metallopeptidase activity
GO:0003774 cytoskeletal motor activity
Molecular Function
Count
5
10
15
20
3.0
3.5
4.0
4.5
5.0
−log10(classicFisher)
GO:1902911 protein kinase complex
GO:1902554 serine/threonine protein kinase complex
GO:0034708 methyltransferase complex
GO:0031970 organelle envelope lumen
GO:0031968 organelle outer membrane
GO:0019867 outer membrane
GO:0015629 actin cytoskeleton
GO:0005758 mitochondrial intermembrane space
GO:0005741 mitochondrial outer membrane
GO:0000307 cyclin−dependent protein kinase holoenzy.
Cellular Component
Count
5.0
7.5
10.0
3.0
3.5
4.0
4.5
−log10(classicFisher)

Supplementary Figure 7. Gene Ontology enrichment analysis for genes with predicted selective sweeps in the Andean gene pool of lima bean.

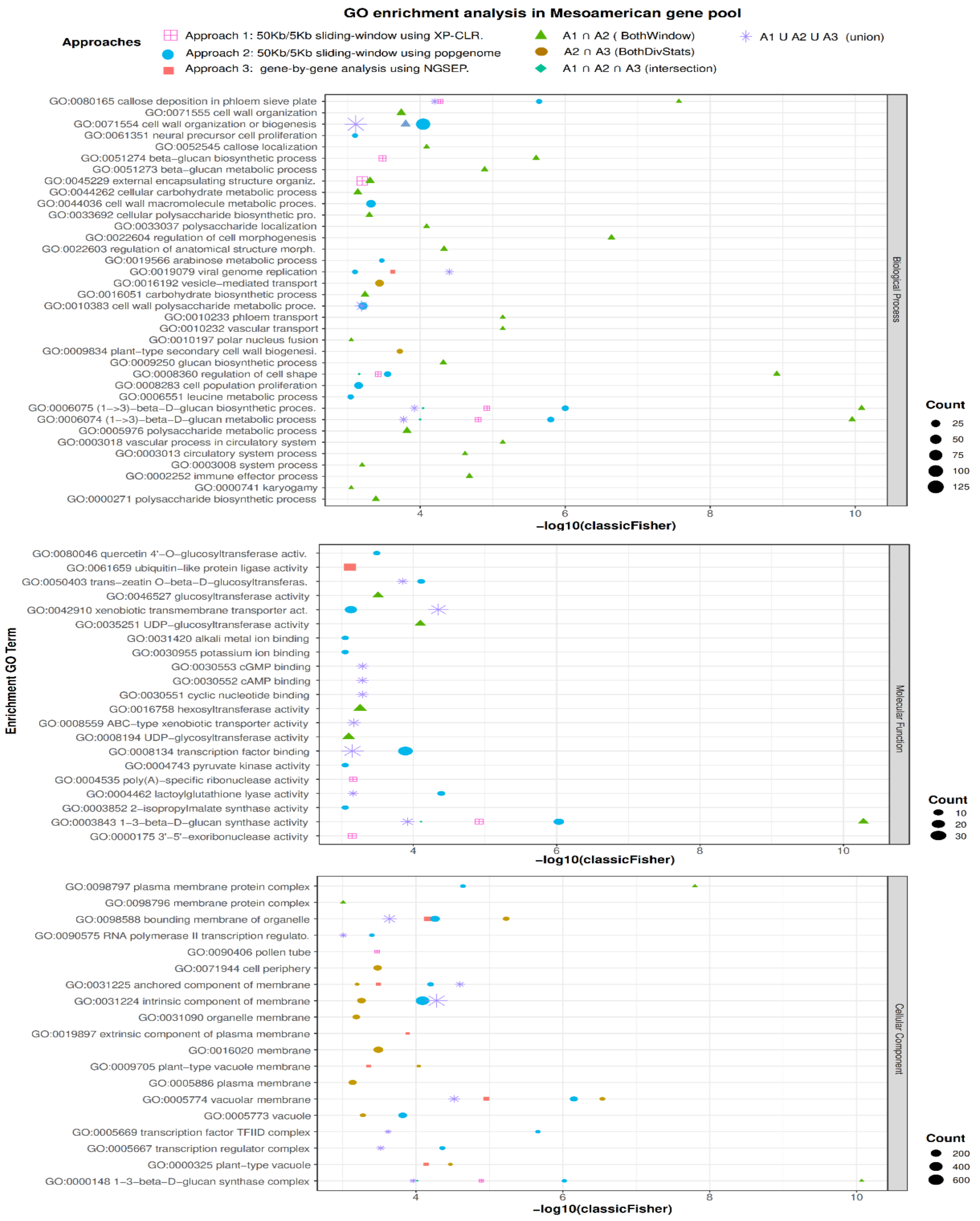

Supplementary Figure 8. Gene Ontology enrichment analysis for genes with predicted selective sweeps in the Mesoamerican gene pool of lima bean.

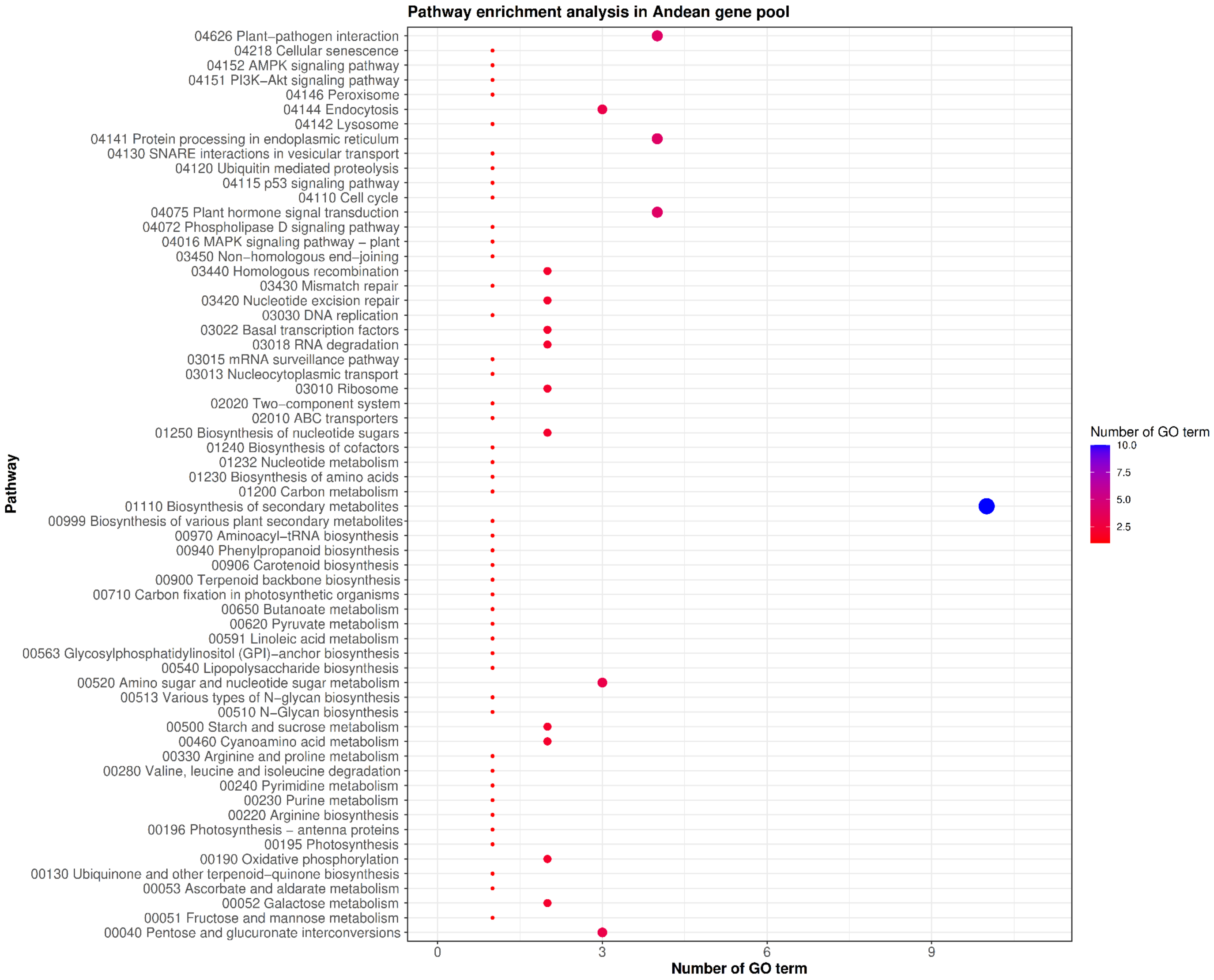

Supplementary Figure 9. Kyoto Encyclopedia of Genes and Genomes (KEGG) pathway analysis for genes with predicted selective sweeps in the Andean gene pool of lima bean.

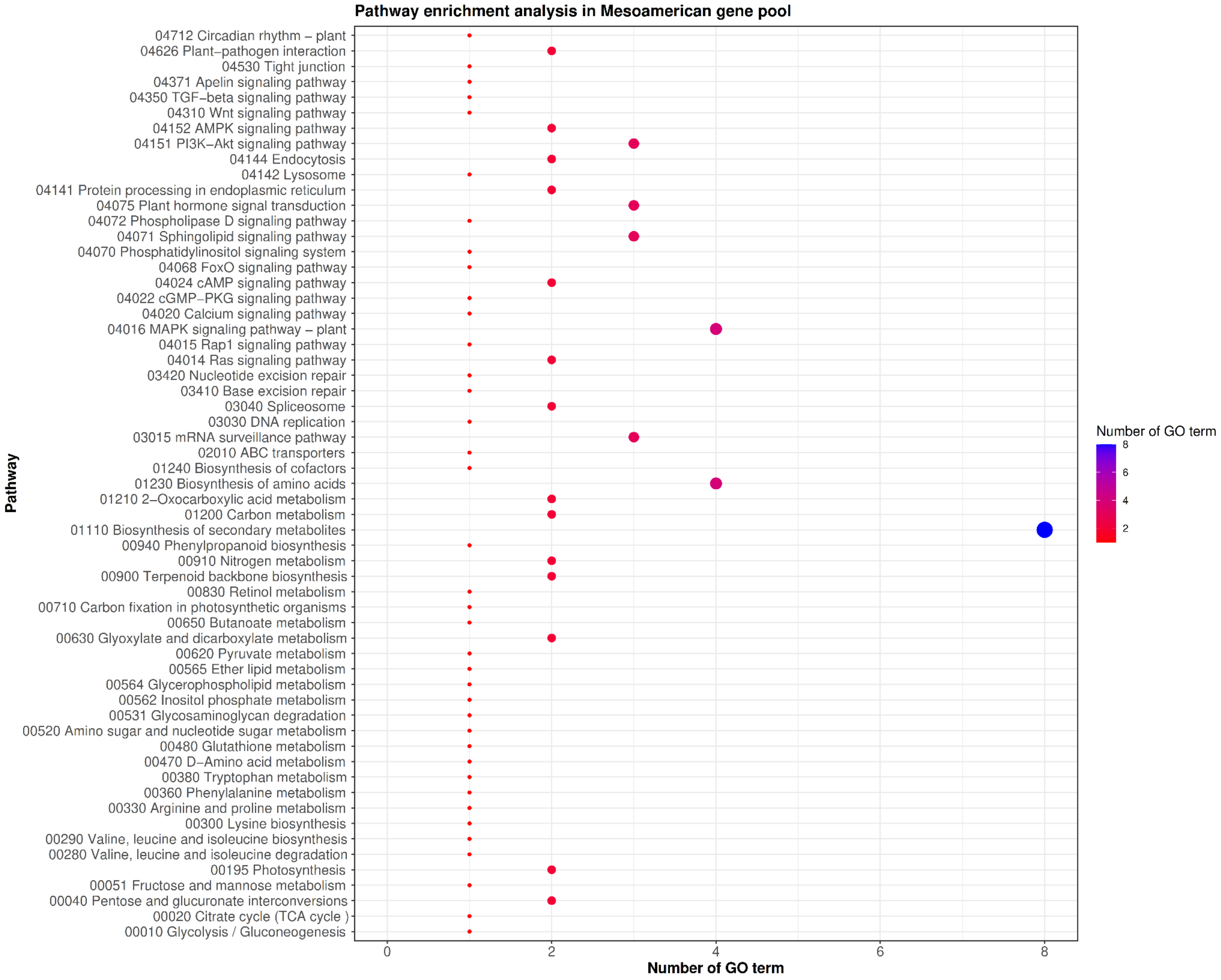

Supplementary Figure 10. Kyoto Encyclopedia of Genes and Genomes (KEGG) pathway analysis for genes with predicted selective sweeps in the Mesoamerican gene pool of lima bean.

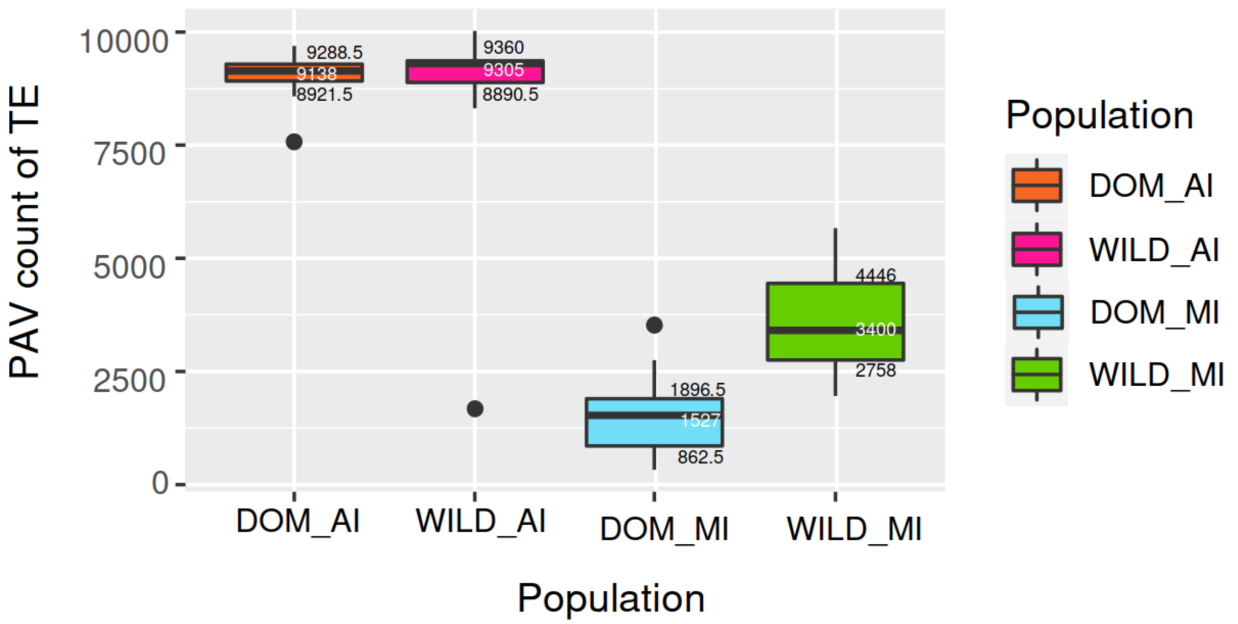

Supplementary Figure 11. Box plot of TE with variation of presence-absence for the 61 sequenced accessions, including the reference accession.

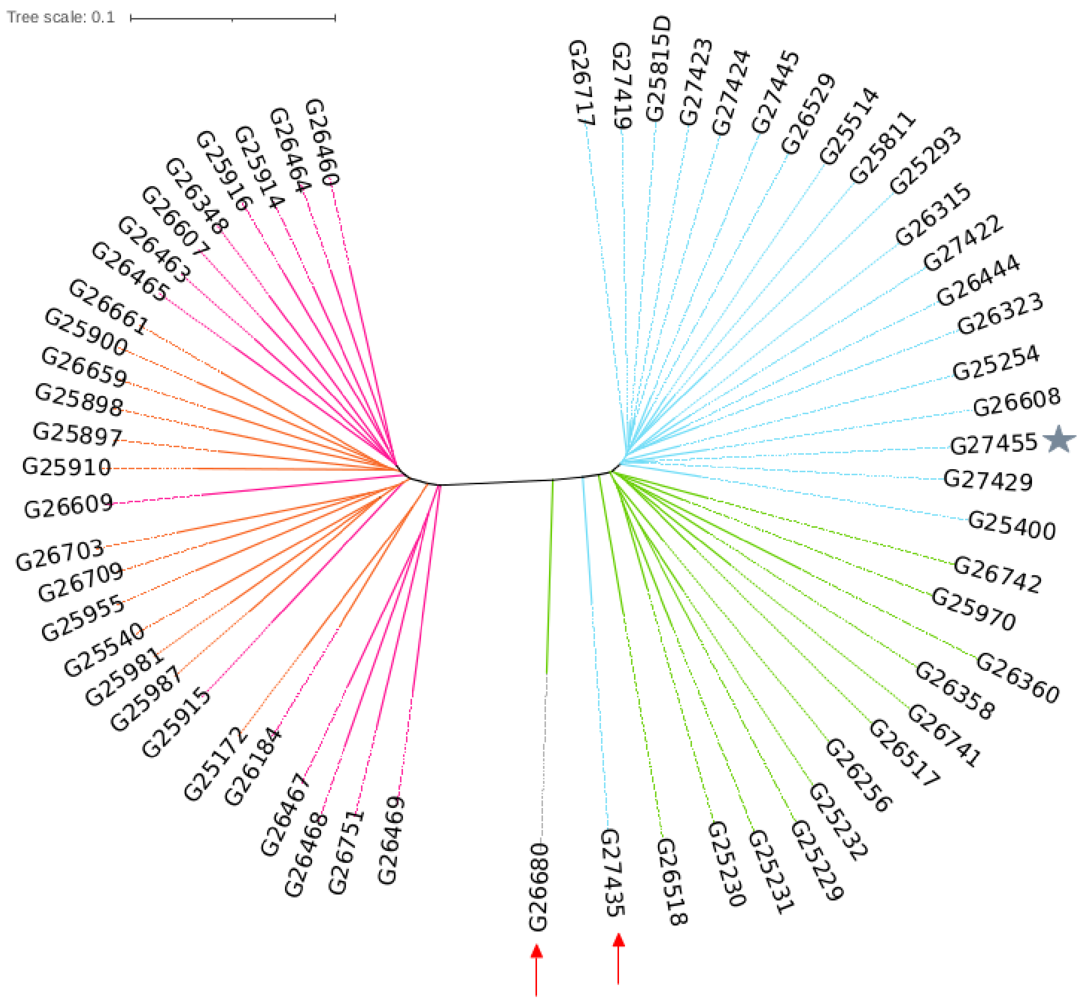

Supplementary Figure 12. Neighbor joining clustering built from the TE presence/absence alleles. Colors differentiate the population of origin for each accession (DOM_AI=domesticated Andean, WILD_AI= wild Andean, DOM_MI= domesticated Mesoamerican, WILD_MI= wild Mesoamerican). The accession highlighted with a red arrow (G27435, G26680) is admixed between Mesoamerican gene pools (MI and MII) and the accession marked with a star corresponds to the reference genome.

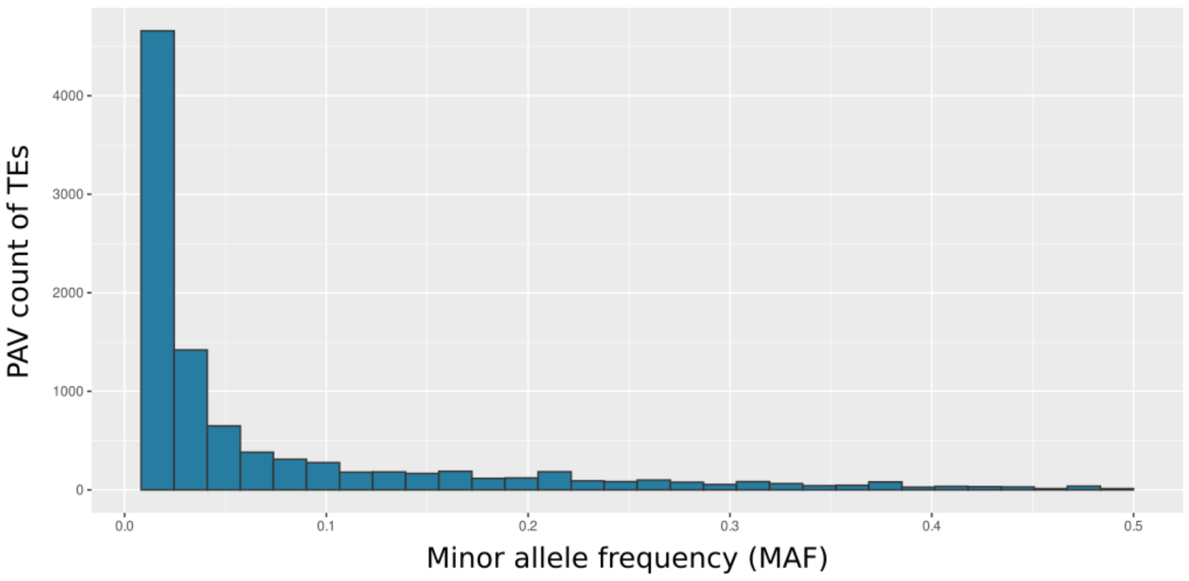

Supplementary Figure 13. Minor allele frequency (MAF) distribution in the PAV transposon dataset. Distribution of MAF for all PAV of TEs in the 61 accessions.

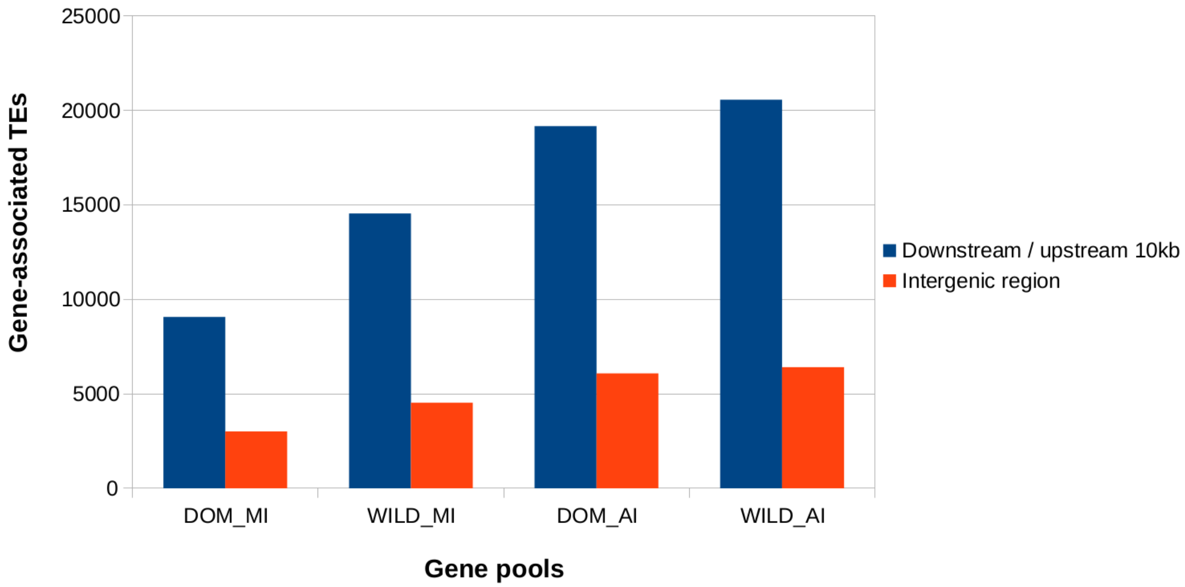

Supplementary Figure 14. Number of TEs with presence/absence variation (PAV) within each lima bean gene pool, discriminated by closeness to a protein coding gene.

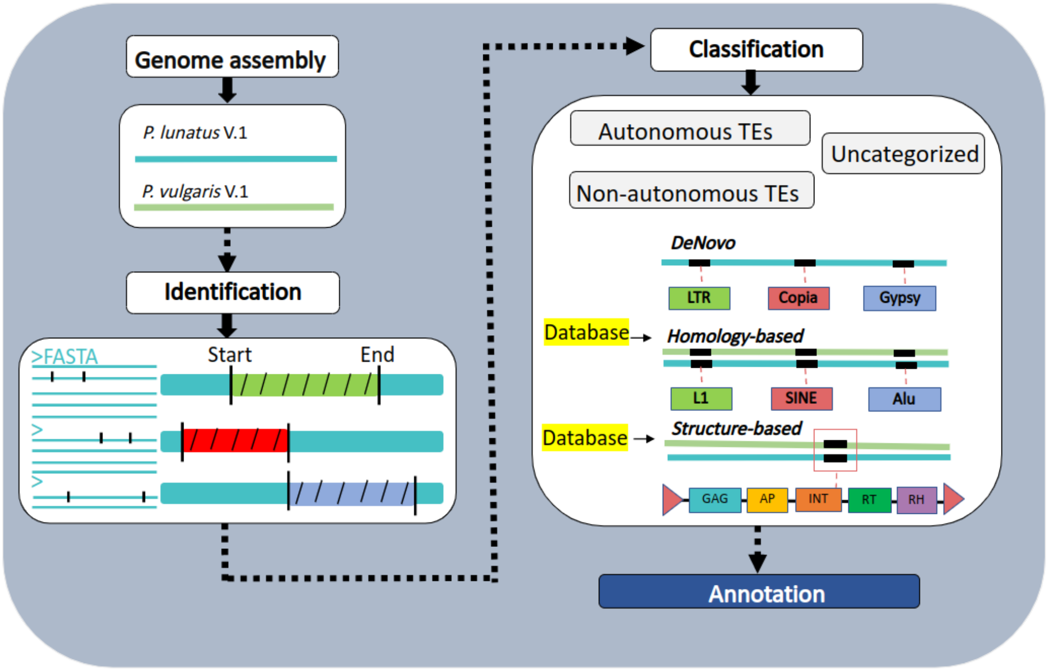

Supplementary Figure 15. Workflow of the analysis carried out to annotate transposable elements from genome assemblies.

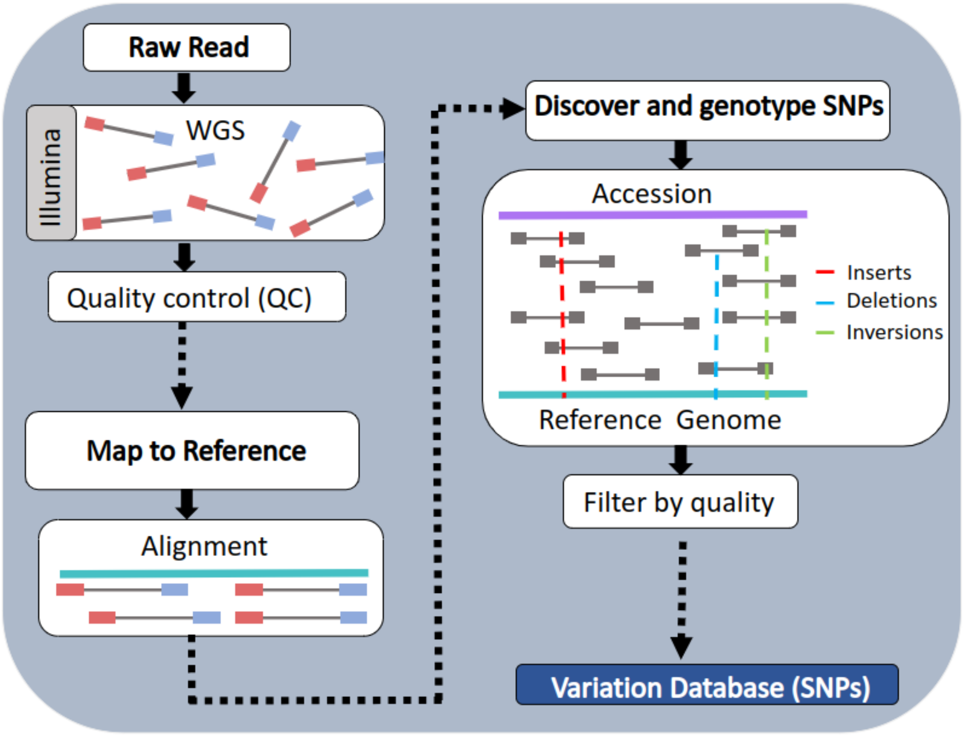

Supplementary Figure 16. Workflow to analyze genetic variability in lima bean from the detection and genotyping of Single Nucleotide Polymorphisms (SNP) in *P. lunatus*.

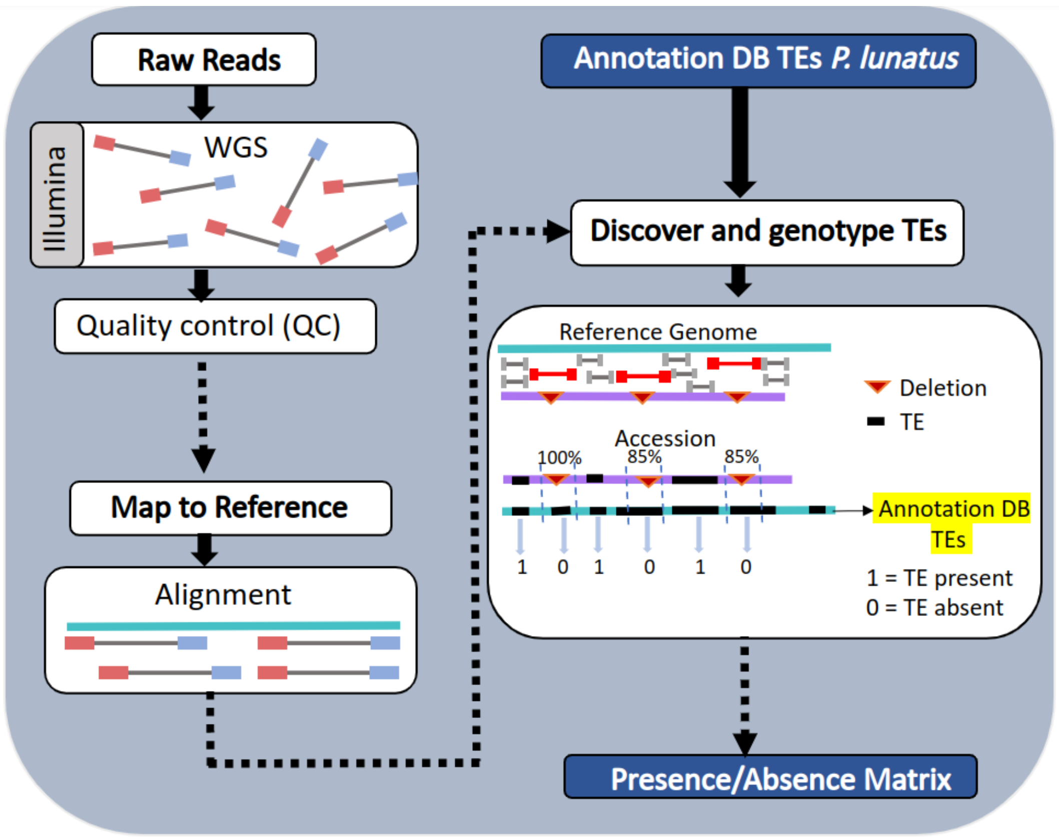

Supplementary Figure 17. Workflow of the analysis of presence absence variability (PAV) TEs.
