# Supplementary material for: Selection signatures and population dynamics of transposable elements in lima bean
Source: Commun Biol. 2023 Aug 2;6:803. doi: 10.1038/s42003-023-05144-y (PMC10397206; doi:10.1038/s42003-023-05144-y)
Supplement: Supplementary file 3 — Description of Additional Supplementary Files [file 42003_2023_5144_MOESM3_ESM.docx]

**Description of Additional Supplementary Files**

**File name:** Supplementary Data 1

**Description:** Database of transposable elements annotated in the lima bean V1 reference genome.

**File name:** Supplementary Data 2

**Description:** Database of transposable elements annotated in the common bean reference genome V1.

**File name:** Supplementary Data 3

**Description:** CIAT seed passport information for the 60 accessions sequenced in this study.

**File name:** Supplementary Data 4

**Description:** Genes observed in the Andean and Mesoamerican gene pools within selective sweeps identified by at least one of three approaches.

**File name:** Supplementary Data 5

**Description:** Matrix of TEs with PAV in lima bean.

**File name:** Supplementary Data 6

**Description:** TEs with PAV that differentiate Andean and Mesoamerican gene pools.

**File name:** Supplementary Data 7

**Description:** TEs with PAV close to genes reported to be important in lima bean domestication.

**File name:** Supplementary Data 8

**Description:** Numeric supporting data for figures
