# Supplementary material for: Selection signatures and population dynamics of transposable elements in lima bean
Source: Commun Biol. 2023 Aug 2;6:803. doi: 10.1038/s42003-023-05144-y (PMC10397206; doi:10.1038/s42003-023-05144-y)
Supplement: Supplementary file 12 — Reporting Summary [file 42003_2023_5144_MOESM12_ESM.pdf]

nature portfolio

Corresponding author(s): Jorge Duitama

Last updated by author(s): Jul 13, 2023

Reporting Summary

Nature Portfolio wishes to improve the reproducibility of the work that we publish. This form provides structure for consistency and transparency in reporting. For further information on Nature Portfolio policies, see our Editorial Policies and the Editorial Policy Checklist.

Statistics

For all statistical analyses, confirm that the following items are present in the figure legend, table legend, main text, or Methods section.

n/a	Confirmed	
☐	☒	The exact sample size (*n*) for each experimental group/condition, given as a discrete number and unit of measurement
☐	☒	A statement on whether measurements were taken from distinct samples or whether the same sample was measured repeatedly
☐	☒	The statistical test(s) used AND whether they are one- or two-sided<br>*Only common tests should be described solely by name; describe more complex techniques in the Methods section.*
☒	☐	A description of all covariates tested
☐	☒	A description of any assumptions or corrections, such as tests of normality and adjustment for multiple comparisons
☐	☒	A full description of the statistical parameters including central tendency (e.g. means) or other basic estimates (e.g. regression coefficient) AND variation (e.g. standard deviation) or associated estimates of uncertainty (e.g. confidence intervals)
☐	☒	For null hypothesis testing, the test statistic (e.g. *F*, *t*, *r*) with confidence intervals, effect sizes, degrees of freedom and *P* value noted<br>*Give P values as exact values whenever suitable.*
☒	☐	For Bayesian analysis, information on the choice of priors and Markov chain Monte Carlo settings
☒	☐	For hierarchical and complex designs, identification of the appropriate level for tests and full reporting of outcomes
☒	☐	Estimates of effect sizes (e.g. Cohen's *d*, Pearson's *r*), indicating how they were calculated

Our web collection on statistics for biologists contains articles on many of the points above.

Software and code

Policy information about availability of computer code

Data collection	Sequencing was performed following a standard protocol for WGS at Macrogen (https://dna.macrogen.com/)
Data analysis	Identification of transposable elements: Inpactor v2, CD-HIT v4.8.1, EDTA v2.0.0, RepeatMasker v4.1.2<br>Phylogenetic reconstruction of LTR transposons: CENSOR v4.2.29, MAFFT v7.475, FastTree v2.1.11<br>Read alignment, variant calling, genotyping and filtering: NGSEP v4.2.1, bowtie2 v2.2.4<br>Selective sweeps: XP-CLR v1.1.2, PopGenome v2.7.5<br>Clustering and statistical analysis: hclust, fisher test and the ComplexHeatmap package available in R v4.1.3

For manuscripts utilizing custom algorithms or software that are central to the research but not yet described in published literature, software must be made available to editors and reviewers. We strongly encourage code deposition in a community repository (e.g. GitHub). See the Nature Portfolio guidelines for submitting code & software for further information.

Data

Policy information about availability of data

All manuscripts must include a data availability statement. This statement should provide the following information, where applicable:

- Accession codes, unique identifiers, or web links for publicly available datasets
- A description of any restrictions on data availability
- For clinical datasets or third party data, please ensure that the statement adheres to our policy

The data used in this study is available at the NCBI sequence read archive (SRA) database (https://www.ncbi.nlm.nih.gov/sra) with bioproject accession number PRJNA596114. The genomic variation database is available at the European Variation Archive (EVA) database under the bioproject accession number PRJEB62157. The reference genome assembly is available at the Assembly database of NCBI (https://www.ncbi.nlm.nih.gov/assembly/) with the accession number GCA_013389735.1. The genome is also available on Phytozome (https://phytozome-next.jgi.doe.gov/). Annotated transposable elements are included as supplementary files of this publication (Supplementary Data 1 and 2). Numerical source data for graphs and charts is available (Supplementary Data 8). All other data are available from the corresponding author (or other sources, as applicable) on reasonable request.

Human research participants

Policy information about studies involving human research participants and Sex and Gender in Research.

Reporting on sex and gender	This study did not involve research with human participants
Population characteristics	N/A
Recruitment	N/A
Ethics oversight	N/A

Note that full information on the approval of the study protocol must also be provided in the manuscript.

Field-specific reporting

Please select the one below that is the best fit for your research. If you are not sure, read the appropriate sections before making your selection.

☒ Life sciences ☐ Behavioural & social sciences ☐ Ecological, evolutionary & environmental sciences

For a reference copy of the document with all sections, see nature.com/documents/nr-reporting-summary-flat.pdf

Life sciences study design

All studies must disclose on these points even when the disclosure is negative.

Sample size	We selected around 15 wild and 15 domesticated individuals from the gene pools MI and AI of Lima bean according to availability at CIAT and taking into account that most cultivated plants belong to these gene pools. For the case of the AI wild population, the sequenced samples are over 90% of the collection available at CIAT. We followed the recommendation given by an anonymous reviewer of a previous work, who stated that 15 individuals per population were sufficient to identify confidently selective sweeps from WGS data. The statement was supported by these manuscripts: https://doi.org/10.1038/ng.715, https://doi.org/10.1038/nbt.2050 and https://doi.org/10.1038/ng.3845
Data exclusions	All sequenced samples were included in the analysis
Replication	Considering the current quality of Illumina sequencing, we did not perform replication of the DNA sequencing experiments. Read mapping was performed using two different software tools, obtaining similar results.
Randomization	Samples were selected based on the gene pool of origin which we assessed based on genotyping-by-sequencing data. The results of that work are available with the publication of the Lima bean genome (https://doi.org/10.1038/s41467-021-20921-1). We applied the relevant statistical tests to identify selective sweeps from this experimental design.
Blinding	We consider that blinding is not applicable in this case.

Reporting for specific materials, systems and methods

We require information from authors about some types of materials, experimental systems and methods used in many studies. Here, indicate whether each material, system or method listed is relevant to your study. If you are not sure if a list item applies to your research, read the appropriate section before selecting a response.

Materials & experimental systems

n/a	Involved in the study	
☒	☐	Antibodies
☒	☐	Eukaryotic cell lines
☒	☐	Palaeontology and archaeology
☒	☐	Animals and other organisms
☒	☐	Clinical data
☒	☐	Dual use research of concern

Methods

n/a	Involved in the study	
☒	☐	ChIP-seq
☒	☐	Flow cytometry
☒	☐	MRI-based neuroimaging
